# Occupational exposure to solar ultraviolet radiation among outdoor workers in Lisbon, 2023—first results of the MEAOW study

**DOI:** 10.3389/fpubh.2025.1659663

**Published:** 2025-10-27

**Authors:** Fernanda Carvalho, Claudine Strehl, Jorge Barroso-Dias, Maria Miguel Castela, Fabriziomaria Gobba, Luís Velez Lapão, Tom Loney, Mélanie Raimundo Maia, Alberto Modenese, Swen Malte John, Ana Rodrigues, Cristina Pinho, Cara Bieck, Thomas Tenkate, Stephan Westerhausen, Marc Wittlich, Marília Silva Paulo

**Affiliations:** ^1^Portuguese Institute for Sea and Atmosphere, Lisbon, Portugal; ^2^Center for Sci-Tech Research in Earth System and Energy - CREATE, IIFA, University of Évora, Évora, Portugal; ^3^Institute for Occupational Safety and Health of the German Social Accidents Insurance (IFA), Hamburg, Germany; ^4^Departamento de Saúde, Higiene e Segurança, Câmara Municipal de Lisboa, Lisbon, Portugal; ^5^Portuguese Society of Occupational Medicine, Working Committee "Work at Open Air", Lisbon, Portugal; ^6^NOVA National School of Public Health, NOVA University Lisbon, Lisbon, Portugal; ^7^Department of Biomedical, Metabolic and Neural Sciences, University of Modena & Reggio Emilia, Modena, Italy; ^8^UNIDEMI, Department of Mechanical and Industrial Engineering, NOVA School of Science and Technology, Universidade NOVA de Lisboa, Lisbon, Portugal; ^9^College of Medicine, Mohammed Bin Rashid University of Medicine and Health Sciences, Dubai Health, Dubai, United Arab Emirates; ^10^WHO Collaborating Centre on Health Workforce Policy and Planning, Global Health and Tropical Medicine, Universidade Nova de Lisboa, Lisbon, Portugal; ^11^Institute for Interdisciplinary Dermatological Prevention and Rehabilitation (iDerm), Osnabrück, Germany; ^12^Department of Dermatology, Environmental Medicine and Health Theory, Institute for Health Research and Education (IGB), Faculty of Human Sciences, Osnabrück University, Osnabrück, Germany; ^13^Division of Occupational Medicine, Department of Occupational Medicine, Hazardous Substances and Health Sciences, German Social Accident Insurance for the Health and Welfare Services, Hamburg, Germany; ^14^CHRC, NOVA Medical School, Faculdade de Ciências Médicas, NMS, FCM, Universidade NOVA de Lisboa, Lisbon, Portugal; ^15^NOVA National School of Public Health, Public Health Research Center, Comprehensive Health Research Center, CHRC, REAL, CCAL, NOVA University Lisbon, Lisbon, Portugal

**Keywords:** personal dosimetry, occupational exposures, outdoor workers, solar UV radiation, ultraviolet radiation exposure

## Abstract

**Introduction:**

Solar ultraviolet radiation (UVR) exposure is the primary external factor associated with the development of skin cancer. Accurate, valid, and reliable objective estimates of individual UVR exposure are required to quantify the risk of skin cancer in outdoor workers. Such data can be used to develop and implement policies and practices to reduce, or at least manage, UVR exposure in outdoor workers. Currently, there is a dearth of objective exposure data for many countries. Lisbon, as a low-mid-latitude region (38°46′ N), experiences a high UV Index (UVI) for a long period of the year, increasing the potential risk of skin cancer among outdoor workers in Portugal. This is the first study to objectively measure personal solar UVR exposure among outdoor workers in Portugal.

**Methods:**

This study used a prospective observational design during seven consecutive months (April to October 2023) studying personal UV exposure of Asphalthers, Gardeners, Gravediggers, Pavers, and Sanitation Workers. Measurements of personal exposure were conducted using the GENESIS-UV measurement system, and ambient solar UVR data was estimated Jm^−2^ utilizing a UV-Biometer radiometer.

**Results:**

Personal hourly and daily doses measured by the GENESIS-UV measurement system were lower than the solar irradiation measured on a horizontal surface by the UV-Biometer radiometer. Gravediggers and Gardeners showed in average, the highest monthly daily averages (250 Jm^−2^ and 266 Jm^−2^, respectively). The maximum monthly daily average occurred for Gravediggers in the month of April (363 Jm^−2^). Pavers recorded the lowest solar UVR average daily doses (62 Jm^−2^). Sanitation Workers recorded the highest average daily dose (837 Jm^−2^, July 7th). The maximum single dosimeter value was accumulated by Gravediggers (1,097 Jm^−2^, May 9th).

**Discussion:**

This study measured solar UVR exposure in important occupations not so often studied. The ICNRIP occupational limit value of 133 J/m^−2^ was surpassed in all occupations except the Pavers. These results showcase that the design of adequate prevention campaigns for preventing occupational skin cancer in outdoor workers should include personalized exposure risk messaging in the future.

## 1 Introduction

Ultraviolet (UV) radiation (UVR) is divided into three main wavelength regimes: UVC (100 nm to 290 nm), UVB (290 nm to 320 nm), and UVA (320 nm to 400 nm) (1, 2). This subdivision arose from standardization and may differ between fields of study (1–3). Hence, due to this fact, international agreements generally establish the boundary between UVA and UVB at 315 nm (1, 2), enabling consistent monitoring and regulation of UVR exposure. The history of UVR research shows an increasing recognition of its effects on human health and ecosystems. Initially identified as a cause of sunburn, subsequent studies have revealed UVR’s role in vitamin D synthesis, its potential to damage deoxyribonucleic acid (DNA), and its action as an antiseptic agent (1, 2). Furthermore, solar UVR has a high potential to inactivate viruses in natural environments (4). These multiple properties continue to drive research, to inform public health policies and environmental protection efforts (1, 5).

### 1.1 Exposure to solar UVR

At the beginning of the 20th century, it was discovered that exposure to sunlight prevented—and in some cases cured—rickets (osteomalacia). While small amounts of UVR are beneficial to human health and essential in the synthesis of vitamin D (i.e., cholecalciferol, also called vitamin D_3_), overexposure to solar UVR can have serious adverse health effects. The main damage is to the skin, eyes, and immune system (5–7). The acute effects of UVR exposure include erythema of the skin and photokeratitis of the eyes. The chronic effects of UVR exposure on the skin include photo-aging and the development of skin cancer, particularly in individuals with fair skin (phototype I and II, according to the Fitzpatrick Scale, a numerical classification scheme taking into account the pigmentation and sun-related behavior of human skin). UVA radiation, which has a pronounced effect on the subcutaneous tissues, is the cause of many chronic degenerative changes to the skin (i.e., photoaging), resulting from its action on various constituent elements of the skin (keratinocytes, melanocytes, collagen, elastin, blood vessels) (1, 7, 8). Skin cancers are mainly caused by long-term, intense and/or cumulative exposure to solar UVR, i.e., the typical exposure of unprotected outdoor workers (9). In 2012, the International Agency for Research on Cancer (IARC) classified solar UVR as a Group 1 carcinogen, acknowledging that approximately 65 to 90% of skin cancer cases are attributable to solar UVR exposure due to biological damage (10–12). Considering the eyes, the main chronic effects include cataracts, pterygium, and rare eye tumors such as ocular melanoma and the squamous cell carcinomas of the cornea and of the conjunctiva (13). Long-term solar UVR exposure has a significant impact in terms of disease burden: it is estimated that approximately 15 million people in the world are blind due to cataracts, of which 10% are due to solar UVR exposure (14). Moreover, non-melanoma skin cancers (NMSC) due to solar UVR exposure are the most frequent malignancies worldwide in individuals with fair skin phototypes, and among the most frequent occupational cancers (15, 16). In Portugal, where fair skin phototypes are prevalent and outdoor occupations such as agriculture and fishing are commonplace, the incidence of skin cancer has been steadily increasing creating a burden for the population and the healthcare services (17, 18). The country’s geographic location and cultural habits, such as prolonged sun exposure during leisure and work, contribute to elevated levels of UVR exposure. According to the EU-OSHA, 14.5 million outdoor workers in the EU are potentially exposed to solar UVR for at least 75% of their working time (19), with Portugal being one of the countries most affected (19–21).

According to the World Health Organization (WHO), everyone, in general, would be at risk of UVR exposure, especially solar UVR, while some people are also exposed to artificial UVR sources (e.g., in medicine, industry and for disinfection and cosmetic purposes) (22). For solar UVR, outdoor workers would be the highest risk group due to their occupational exposure, along with children and adults with high recreational solar UVR exposure (1, 5, 23, 24). The International Labour Organization (ILO) also addresses the issue, as a workplace hazard, focusing on every occupational setting that deals with UVR, including the sun as a source (25). However, existing guidelines are principally oriented toward the prevention of acute effects such as photo-keratitis and sunburn (2, 26), while long-term risks remain inadequately addressed. The current occupational exposure limit of 30 J/m^2^ per 8-h shift applies to both solar and artificial UVR (Directive 2006/25/EC) (27). This limit poses challenges for accurate outdoor assessment and fails to account for chronic exposure effects (27), and a regulatory gap that emphasizes the pressing need for comprehensive protective strategies, particularly in high-risk countries such as Portugal.

### 1.2 UVR climatology for Lisbon

**Figure 1:**
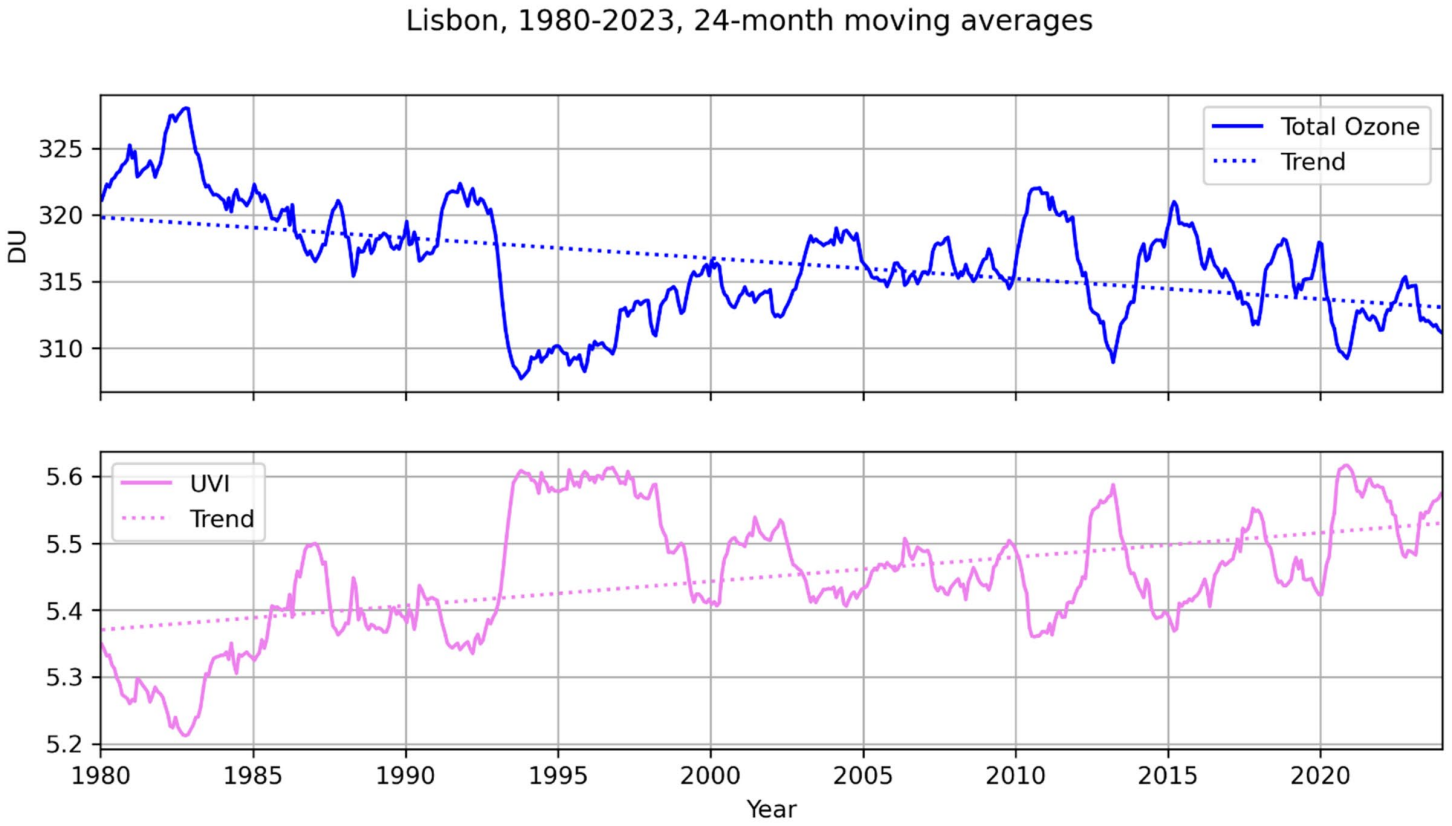
12-month moving averages of clear sky UVI and O_3_ layer thickness obtained in Lisbon by various satellites since 1980 from the tropospheric emission monitoring internet system (TEMIS).

The Earth’s atmosphere is composed of layers defined by vertical temperature gradients. The layers of the atmosphere are the troposphere, stratosphere, mesosphere, thermosphere and exosphere. During climate change, the conditions within our atmosphere are changing. However, the effects vary across the different atmospheric layers. The troposphere, extending from the Earth’s surface to altitudes between 10 km and 20 km, is characterized by a decrease in temperature with increasing height. Additionally, this atmospheric layer is distinguished by dynamic vertical wind motion, appreciable water vapor, and a variety of weather phenomena. This is also the region where the absorption of long-wavelength radiation by greenhouse gases (GHGs) occurs. Due to the increase in GHGs (i.e., global warming), this layer will be mainly responsible for the cooling of the stratosphere during the second half of the 21st century (28). The stratosphere, which is situated above the troposphere, extends up to approximately 50 kilometers, and owes its existence to the heating of ozone by solar UVR. Its temperature varies from −85 °C or less near its upper boundary to roughly 0 °C; however, it has been undergoing a period of cooling since the 1980s. This decline in temperature is mainly due to the decrease in stratospheric ozone (O_3_), driven by the presence of chlorofluorocarbons. As these O_3_-damaging substances disappear from the stratosphere and show signs of recovery, the radiative cooling of the stratosphere due to the warming of the troposphere can lead to a slower recovery. Ozone plays a critical role by absorbing solar UVR, and as harmful substances are phased out and the O_3_ layer begins to recover, the influence of tropospheric warming on stratospheric cooling may become more pronounced (28). Over the last 60 years, the temperature of the stratosphere over Portugal has decreased by between 1.2 and 1.5 °K, mainly due to the reduction of O_3_ in the stratosphere (Figure 1). Additionally, long-term trends indicate a small but statistically significant decrease in total ozone (−0.48 ± 0.04% per decade) and a rise in UV index (UVI; +0.67 ± 0.05% per decade).

The observed increase in ultraviolet radiation at the surface can be explained by the decrease in the total amount of O_3_ (29), especially in Portugal until 1994. However, despite some evidences from measurements at some stations in the world, no statistically significant decreases in UV-B radiation due to the beginning of the ozone recovery have yet been detected (30). On the other hand, there is evidence that policies to reduce particulate emissions have improved the quality and made the atmosphere more transparent to UVR (31). At the same time, climate change has contributed to an expansion of the North Atlantic subtropical anticyclone, thus decreasing the conditions for cloud formation and increasing solar radiation at the surface (32).

**Figure 2:**
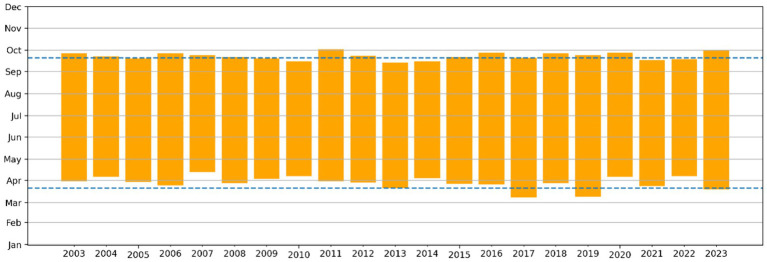
Annual seasons from 2003 to 2023 for clear sky with “high” UV index (UVI ≥ 6) estimated for Lisbon by TEMIS service. Dashed lines indicate the average dates for the equinoxes (March 21st and September 20th).

Another important aspect is the annual total number of days with UVI ≥ 6, i.e., high to extreme, according to the WHO and World Meteorological Organization (WMO) classification. Although no trend can be discerned in the periods when the UVI is considered “high” (≥ 6), in Lisbon, there are more than 150 days a year with high UVI (Figure 2).

For the prevention of occupational exposure to solar UVR, since 2016, the WHO promoted a new project to provide joint estimates of cataract and skin cancer burden due to occupational solar UVR exposure (24, 33). These estimates have been recently published for non-melanoma skin cancers (34), calculating a global burden of approximately 19,000 deaths and 0.5 million DALYs per year on a population of 1.6 billion exposed workers in the world (34). Despite this estimated high burden of occupational disease, to the best of our knowledge, there have been no previous efforts in Portugal to conduct a national research initiative aimed at assessing the real-time exposure of outdoor workers and promoting a multi-dimensional preventive intervention to reduce their UV-related health risks.

## 2 Materials and methods

### 2.1 Study design

This study is part of the first stage of an observational prospective study entitled “Digitally measuring solar ultraviolet radiation in outdoor workers,” also known as the MEAOW project from “MEAsuring Outdoor Workers.” A protocol for this study, in which the project is explained in detail, has been published before (35).

### 2.2 Setting

The Lisbon Municipality (CML) has 10,388 indoor and outdoor workers, spread across approximately 400 work institutions and activities, comprising a diverse range of over 65 occupational groups.

### 2.3 Participants

Overall, 91 outdoor workers from Lisbon Municipality were fitted with personal electronic dosimeters to objectively estimate solar UVR during their working hours. The electronic dosimeters were rotated weekly within the teams of outdoor workers from different occupational groups during the 7 months of data collection. In some teams (i.e., the pavers, gravediggers, and sailors), the same person used the dosimeter during the entire seven-month exposure assessment period.

The geographic locations and distribution of outdoor workers were:

- Gardeners—Avenida de Ceuta (*n* = 14);
- Gardeners—Jardim do Campo Grande (*n* = 4);
- Sanitation workers—all city (*n* = 25);
- Asphalters—all city (*n* = 8);
- Pavers—all city (*n* = 6);
- Gravediggers—Alto de São João (*n* = 19);
- Gravediggers—Benfica (*n* = 8);
- Gravediggers—Prazeres (*n* = 5);
- Sailors—Tagus River (*n* = 2).

In Portugal, outdoor workers such as gravediggers, gardeners, pavers, asphalters, sanitation workers, and sailors perform essential roles that facilitate urban functionality and community safety. Those engaged in the maintenance of cemeteries and green spaces, such as gravediggers and gardeners, are frequently exposed to, among other things, UVR. Pavers and asphalters are responsible for the construction and maintenance of roads, often handling heavy, potentially harmful materials in variable weather conditions. Sanitation workers are responsible for cleaning public spaces and collecting waste. Regarding this occupational group, it is important to note that while many of these workers operate during daylight hours, some teams can be assigned to nighttime shifts, thereby significantly reducing their exposure to solar UVR. Conversely, sailors part of the Lisbon Municipality generally undertake seasonal operations, undertaking voyages exclusively when reservations are confirmed, thereby ensuring consistent exposure to outdoor conditions throughout the year.

Two sailors volunteered to participate in the study, but at the end just one performed measurements with the provided measurement equipment. At the end of the measurement period, only 7 measurement days could be classified as valid for this occupation. For this reason, the results are not presented as they are not statistically reliable.

### 2.4 Procedures

The results of the study presented here were collected through a personal electronic dosimetry measurements campaign in a cohort of outdoor workers occupationally exposed to solar UVR. The measurements were performed using the GENESIS-UV (GENeration and Extraction System for Individual Exposure) measurement system (36). Data acquisition was carried out by person-worn electronic data logging dosimeters (X-2012-10, Gigahertz, Turkenfeld, Germany) comprising two sensors to log the UV irradiance in the UVA and UVB/C regimes separately. The erythemal weighting according to S_er_ was achieved by on-device filter disks (37). The data recording interval was set to 1 s, and all cumulative data were derived by summation.

The sensors of the GENESIS UV measuring device (dosimeter) are regularly checked for their calibration status by the manufacturer and if necessary are then calibrated in accordance with national standards. In addition, deviations due to technical aging processes are determined by means of an internal calibration measurement at the beginning and at the end of the measurement period and, if necessary, subsequently corrected in the measured values. If a change of more than 30% has occurred over the years, the dosimeter is technically checked and calibrated by the manufacturer.

The local research team (MRM, JBD, LVL, CP, AR, MSP) was trained on how to prepare and fit the dosimeters for data collection and deal with possible challenges. Two researchers from the GENESIS-UV technical team traveled to Lisbon to provide this scientific training (CS and SW).

Education and training were provided by the researchers’ team to the outdoor workers. Specifically, a training session explained the rationale for the project and the necessary procedures needed from them (i.e., how to wear the dosimeters correctly and how to use the GENESIS-UV data transfer routine). In this session, the occupation supervisors and outdoor workers were present and provided their informed consent to participate in the study.

The research team was also present at the first data transfer of each dosimeter to ensure that the data was transferred correctly and that the participant was confident in the process. During the data collection period, research staff from the local research team visited the outdoor workers and the sites once a month to ensure direct follow-up and engagement of the outdoor workers. Weekly phone calls were also made to the outdoor workers supervisor to ensure a smooth data collection process. Regular data check-ups were then made by the GENESIS-UV team in Germany to ensure proper data transmission.

Data examination was conducted to ascertain the reasons behind the absence of valid data in certain dosimeters. It was verified if the absence of certain data entries could be primarily attributed to device-related issues, or if it could be attributed to human errors—more specifically, to errors in the utilization of the dosimeter by the outdoor workers themselves. The GENESIS-UV team conducted this verification process to ensure the highest possible level of data validity.

### 2.5 Measurements

**Figure 3:**
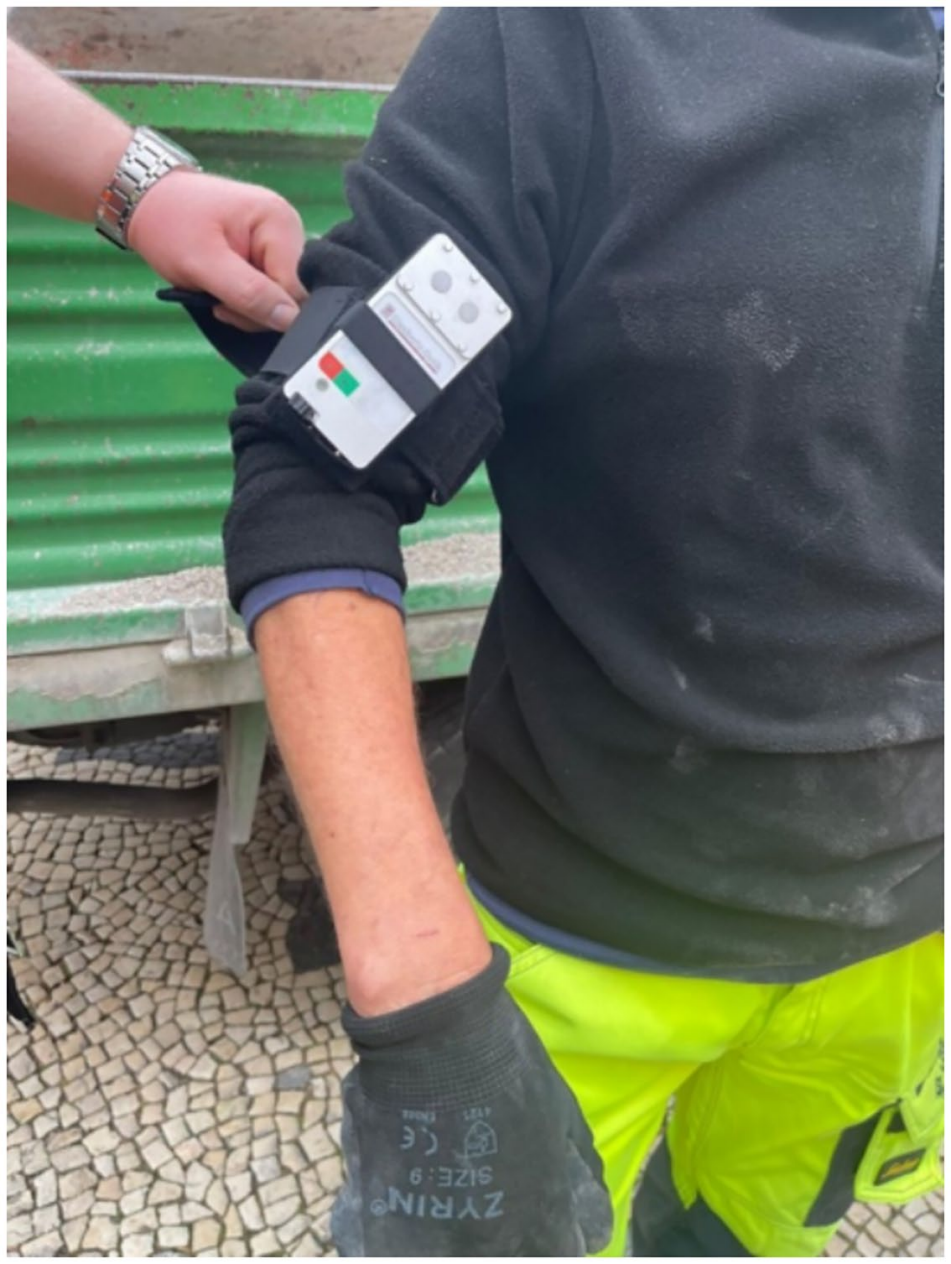
Outdoor workers using the GENESIS-UV data logger dosimeter.

Forty-one GENESIS-UV data logger dosimeters were assigned to outdoor workers from Lisbon Municipality to be used during their occupational activities. The participants were meant to wear the dosimeters every working day during their regular working hours for a measurement period starting at the beginning of April until the end of October. It is assumed that during this period the majority of annual UVR exposure occurs. During the measurement period, from the 10th of April to the end of October 2023, volunteers usually attached the dosimeter to their upper left arm by the aid of a specially designed strap system (Figure 3).

The collected data was later analyzed by the GENESIS-UV team. To guarantee data validity with respect to the proper wearing of the dosimeter, a calibrated accelerometer is integrated within the dosimeter. This accelerometer exhibited a high degree of sensitivity to motion. The purpose of the device was to detect even the most subtle vibrations and movements, thereby serving as evidence that the dosimeters were being actively worn by the outdoor workers during their day of work. In the event of the dosimeter being positioned in a static position on a surface, the sensor would register negligible movement, thus indicating improper usage of the device.

In the process of postprocessing measurement data a visualized daily exposure curve is used to visually inspect every measurement day for plausibility and malfunctions. Plausible data was marked and became the basis for later evaluation (valid measurement days).

### 2.6 Statistical methods

Descriptive statistical techniques were applied to describe the data from the dosimeters. All valid measurement days were included in the further analysis. Based on that monthly daily averages as well as the corresponding standard deviation was calculated for each measurement month (April to October). Furthermore, average daily doses as well as half-hour values were aggregated. An analysis and comparison of the two sets of data—personal occupational solar UVR exposure and measurement data extracted from a UVR biometer from the headquarters of Portuguese Institute of Sea and Atmosphere in Lisbon (nearby the International Airport)—was performed for the purpose of this paper. The UV detector used here (UV-Biometer) is a broadband UV radiometer, model SL-501, manufactured by Solar Light, with a spectral response close to the CIE Erythema action spectrum, with 99.503% of the total response between 280 nm and 320 nm and 0.497% between 320 nm and 400 nm, cosine response error within 5%, nominal response time of 1 s, temperature correction of 1% per K and estimated daily uncertainty of +/− 5%. The measurement data output is in units of UVI. To be able to directly compare measurement data collected by dosimeters to the UV biometer data Exposure Ratio To Ambient (ERTA) values were calculated on several levels and with different key aspects (38). The ERTA defines the ratio between personal (measurement data of dosimeters UV_pers_) and ambient (UVR biometer data over the same exposure time period UV_amb_) UV exposure and is expressed as percentage (Equation 1):


(1) $$ERTA=\frac{UV_{pers}}{UV_{amb}}*100\%$$

For this purpose the data output of the biometer has to be transferred from UVI to erythemally weighted irradiation in Jm^−2^ (39). This can be done by the formula defining the conversion from UVI to erythemally weighted irradiation (22):


$$1\;UVI=0,25\;\frac{mW_{ery}}{m^{2.}}$$

After this conversion UVI measurement data can be aggregated to hourly, daily or monthly averages.

## 3 Results

### 3.1 UV-biometer data

**Figure 4:**
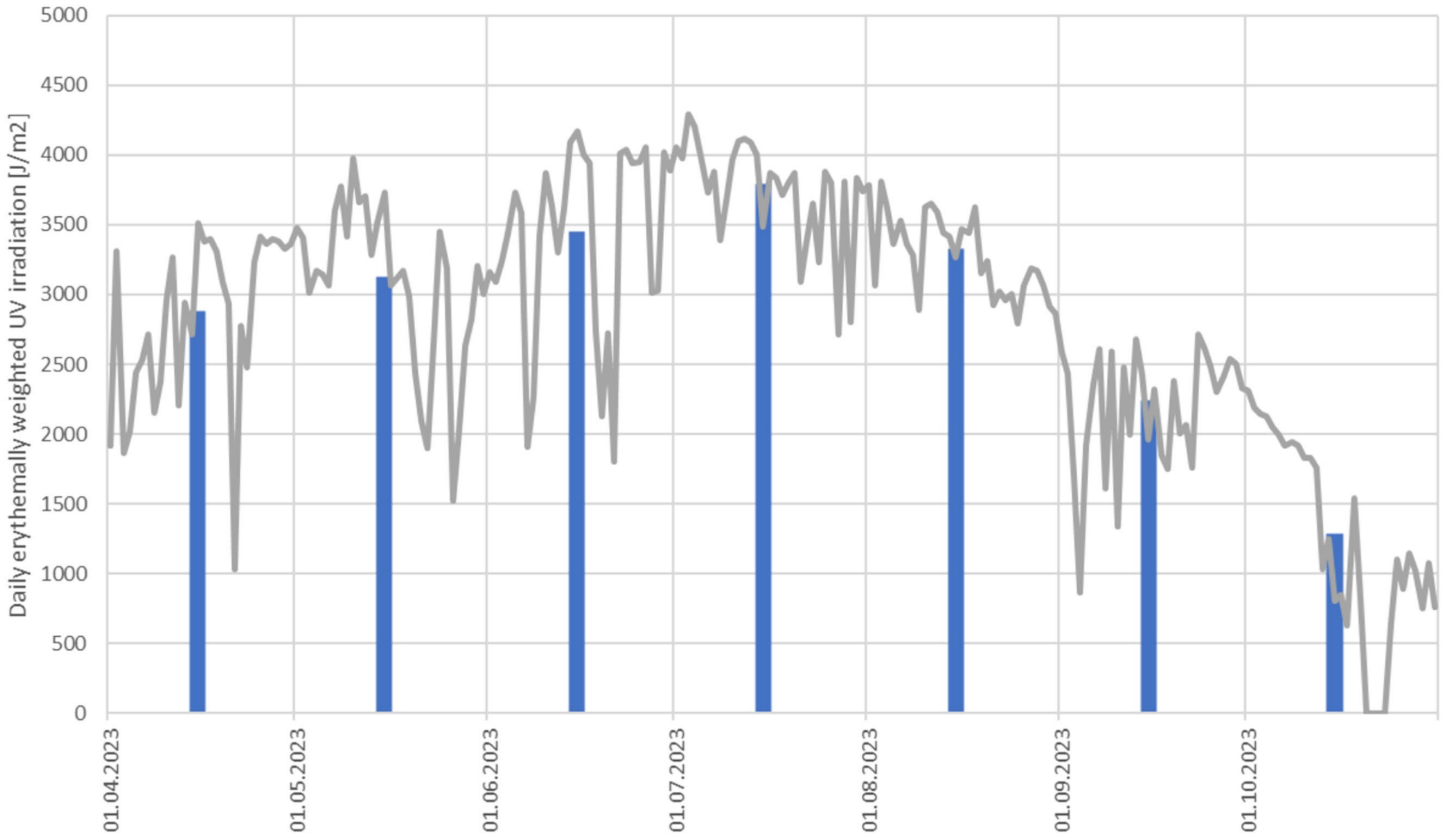
UVR biometer data, daily averages (gray line) and monthly daily averages (blue bars) in units of [Jm^−2^] of erythemally weighted UVR doses.

Figure 4 shows the measurement results for the UVR biometer for the measurement period from April to October. Measurement data were first aggregated to a total daily UVR dose (gray line). For each month, a monthly daily average was calculated (blue bars). The course of UVR measured by the UVR Biometer showed the expected behavior with maximum doses in July. There was a short period of 5 days in October where no measurement data was collected by the UVR biometer (gray curve is dropping to 0). The values measured in our study provide only a very limited insight into the UV climatology in Portugal. Generally, countries located close to the equator experience less annual variation in UV intensity because the solar angle changes less compared to countries situated at higher latitudes.

### 3.2 Monthly daily averages of UVR doses

**Figure 5:**
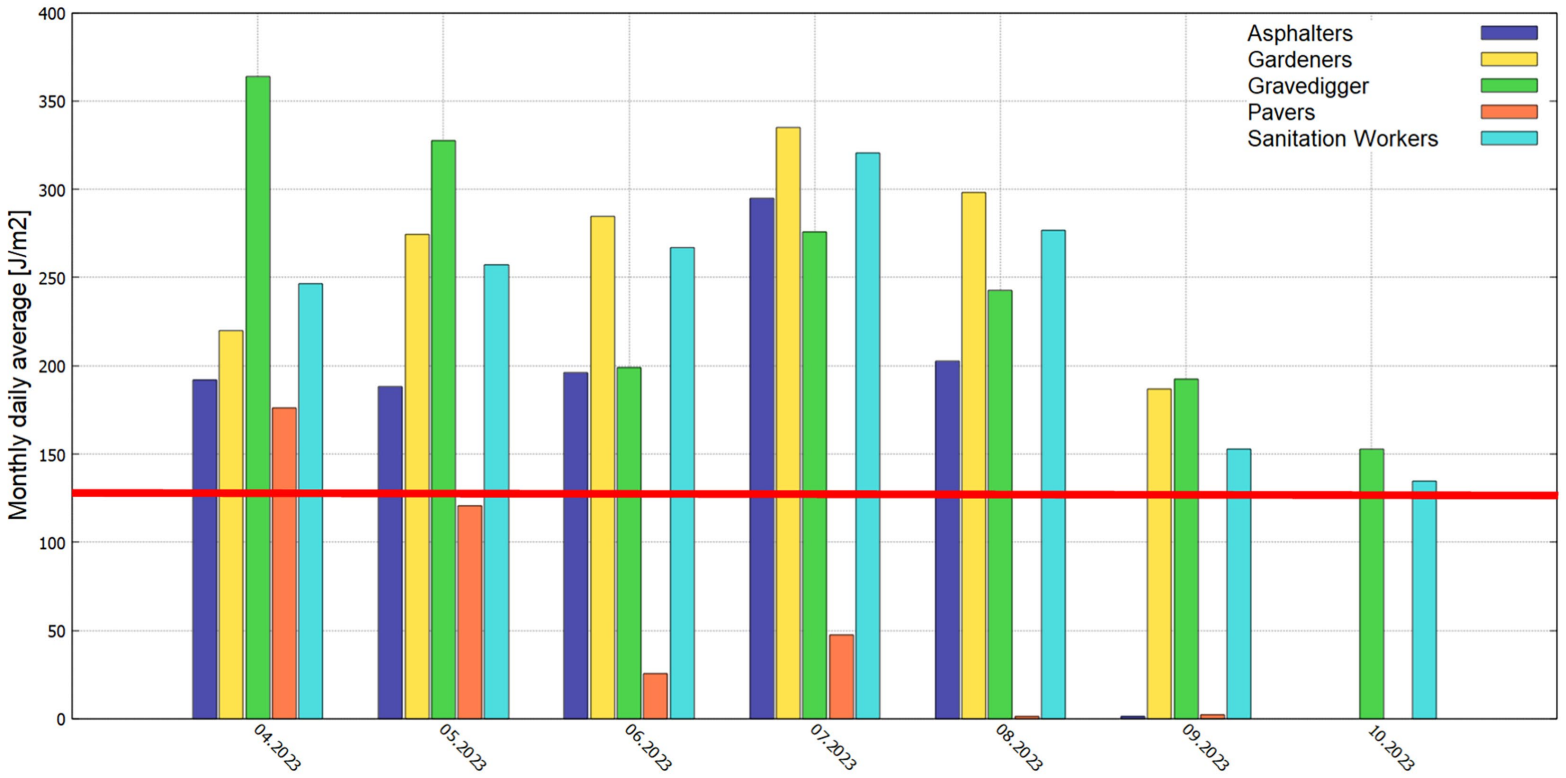
Monthly daily average UV doses [Jm^−2^] for each occupation, the red line indicates the threshold limit of 133 Jm^−2^ as ICNIRP recommendation for a daily limit value.

Monthly daily averages allow to see variations in UVR exposure across the measurement period. Furthermore, they allow to identify occupational exposure patterns across the seasons. Figure 5 visualizes the monthly daily averages of measured UVR doses per occupation for the measurement months April to October. The numerical values of monthly daily averages as well as the related standard deviation and number of corresponding valid measurement days are summarized in Supplementary Table 1. In Supplementary Table 1 entrys are marked in yellow if they show only small statistical validity due to only small numbers of valid measurement days in that month (less than 5). The monthly daily average UVR dose was calculated as mean value over all valid measurement days in the relevant month. Missing data bars in Figure 5 do not indicate missing UVR exposure but rather indicate missing measurement data for the corresponding month.

From Figure 5 it can be seen that each occupation shows an individual exposure profile: some occupations (Gravedigger, Pavers) show high exposure at the beginning of the measurement period (April and May) followed by a small decrease and again higher exposure in the summer time (July). The other occupations show lower exposure at the beginning of the measurement period with constantly rising exposure levels until the maximum exposure level is reached in July (Asphalters, Gardeners, Sanitation Workers), following the course of UV-intensity during the seasons. The Gravedigger and Gardeners showed in average (mean value across all monthly daily averages) the highest monthly daily averages (243 Jm^−2^ resp. 266 Jm^−2^), whereas Pavers showed the lowest (62 Jm^−2^). The maximum monthly daily average occurred for Gravedigger in April (363 Jm^−2^). The ICNRIP occupational limit of 133 Jm^−2^ as maximum daily dose (indicated as red line in Figure 5) is surpassed for almost all occupations in all measurement months, except Pavers that was just above in April. Supplementary Table 1 presents these results.

As previously mentioned, the ERTA defines the ratio between personal and ambient UV exposure (see Equation 1). When dosimeter data is directly compared to UV biometer data (Table 1) it can be seen that, for most of the occupations, the ERTA values are in the range of 5 to 10% which corresponds well to earlier measurements published by Godar et al. (40). Pavers showed the lowest ERTA values with a mean value over all measurement months of 2% and a maximum in April with 6%. Gravedigger had the highest mean ERTA (9.1%) on a monthly basis as well as the highest maximum ERTA of 12.9% in April. Corresponding to monthly daily average doses in Figure 5 each of the occupational profiles shows its specific characteristics. Gravediggers and Pavers show the highest ERTA values in April, whereas Asphalters show the highest ERTA in July, Gardeners in August and Sanitation Workers in October. For Gardeners ERTA values seem to be almost equally distributed across the different months, possibly indicating only little variations in occupational tasks with focus on personal UVR exposure across the year. This is the same for Sanitation Workers. For Gravediggers highest ERTA values occur at the beginning and at the end of the measurement period while these values are the lowest during summer. This could indicate that during the summer, when temperatures are quite high, the employees were able to relocate some of their occupational tasks into more shadowed areas.

**Table 1:** Monthly daily average ERTA-values on the basis of monthly daily averages for all occupations, as well as mean ERTA value over all measurement months (April to October).

Occupation	Monthly daily averages ERTA [%]							ERTA Mean [%]
	April	May	June	July	August	September	October	
Asphalters	6,8	6,1	5,8	7,9	6,2	0,1	-	5,5
Gardeners	7,8	8,9	8,4	8,9	9,1	8,6	-	8,6
Gravediggers	12,9	10,7	5,9	7,4	5,9	8,8	12,3	9,1
Pavers	6,2	3,9	0,8	1,3	0,05	0,1	-	2,1
Sanitation workers	8,7	8,4	7,9	8,6	8,4	7,0	10,9	8,6

### 3.3 Average daily doses

**Figure 6:**
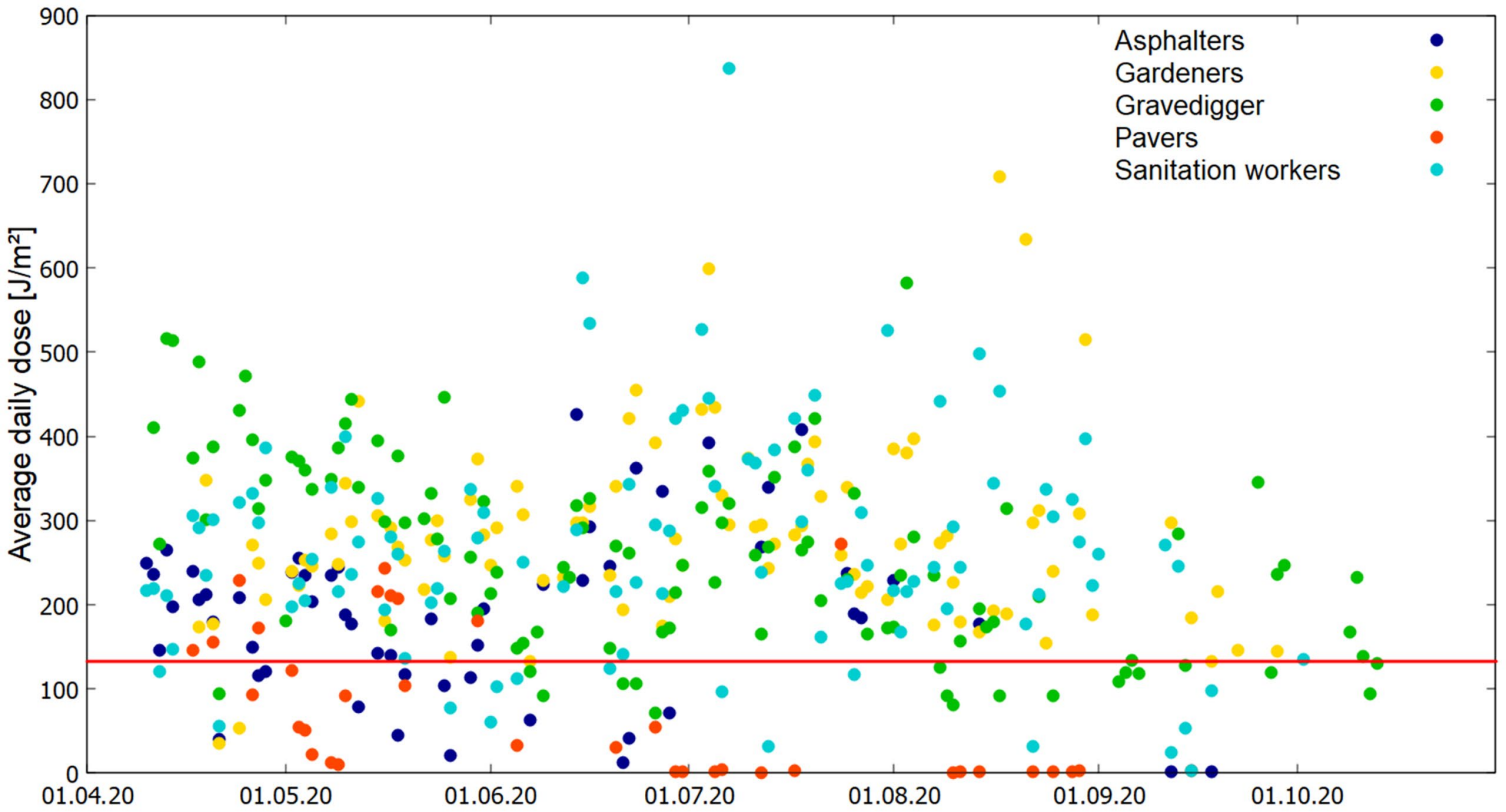
Average daily dose [Jm^−2^] for all measurement days with valid data entries for all occupations. The red line indicates the threshold limit of 133 Jm^−2^ as recommendation for a daily limit value.

Average daily doses allow to identify variations across the single measurement days. As some of the measurement days are comprised of several data sets coming from several participants of one occupation, daily averages were calculated as mean values over all valid data entries for each day of the measurements. Figure 6 shows the distribution of daily averages for each occupation in the course of measurements from April to October.

Sanitation Workers had the highest daily average (837 Jm^−2^, July 7th). The maximum single dosimeter value was recorded by Gravediggers (1,097 Jm^−2^, on May 9th). As is evident in the data, the majority of occupations demonstrate higher daily averages during the summer months (mainly Sanitation Workers and Gardeners), except for those employed as pavers, who exhibit significantly lower values during this period. It may be hypothesized that Pavers have the opportunity/ possibility to remain in shaded areas during their occupational duties. Nonetheless, in addition to the monthly daily averages, the ICNRIP occupational limit of 133 Jm^−2^ as the maximum daily dose (illustrated as the red line in Figure 6) is surpassed on 80% of the measurement days.

**Figure 7:**
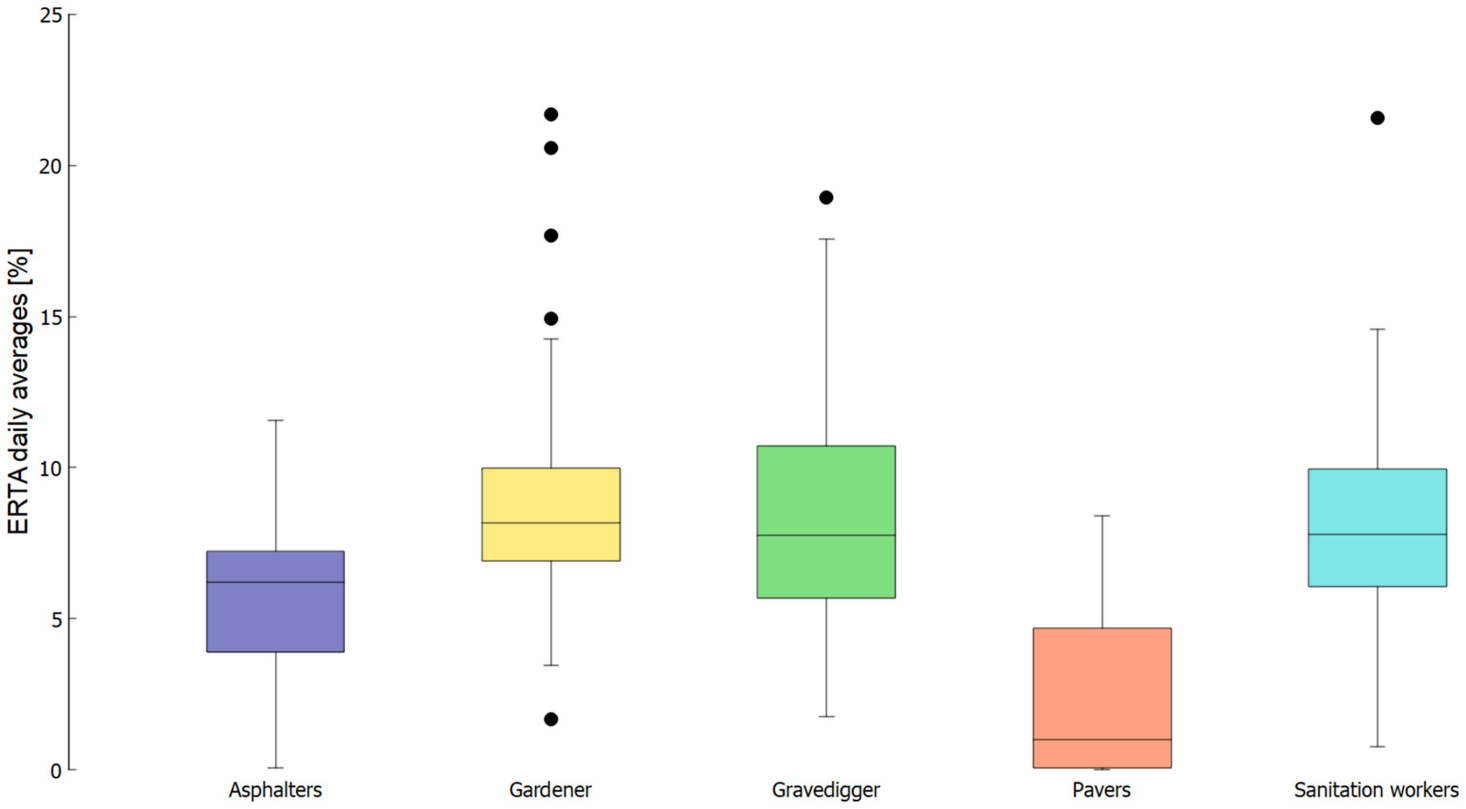
ERTA values [%] on the basis of daily averages doses, box plot for the five investigated occupations.

ERTA was also calculated on the basis of daily averages. Figure 7 visualizes the results in a box plot. Table 2 additionally summarizes the mean and maximum ERTA values on the basis of daily averages. Gardeners show the highest mean value for ERTA (8.62%), as well as the highest maximum ERTA (21.67%). The months of maximum ERTA values on the basis of daily averages slightly varies to data evaluation on the basis of monthly daily averages. Here for most of the occupations the maximum ERTA values are found in the summer months (June, July, August). Only for Gravediggers the maximum ERTA value is found in April, which corresponds to the monthly daily average results. It has to be stated that the course of results in terms of mean and maximum values for daily doses and ERTA values do not have to be necessarily the same as ERTA values depend on the intensity of ambient UVR, which also varies between the single measurement days.

**Table 2:** Mean and maximum (Max) ERTA values [%] on the basis of daily average doses for all occupations, as well as the month of occurrence of the maximum ERTA value.

Occupation	Mean ERTA [%]	Max ERTA [%]	Month of max ERTA
Asphalters	5,72	11,56	June
Gardeners	8,62	21,67	August
Gravediggers	8,37	18,93	April
Pavers	2,33	8,41	July
Sanitation workers	7,98	21,56	July

Gravediggers and Pavers seem to have the highest variability between different measurement days, visible in the larger size of the boxes in Figure 7, indicating possibly varying occupational tasks in terms of UVR exposure. While for Gardeners, Gravediggers and Sanitation Workers daily ERTA values are in the range of 6 to 11%, for Asphalters they are around 4 to 7% and for Pavers they are up to 5%.

### 3.4 Half-hour doses

**Figure 8:**
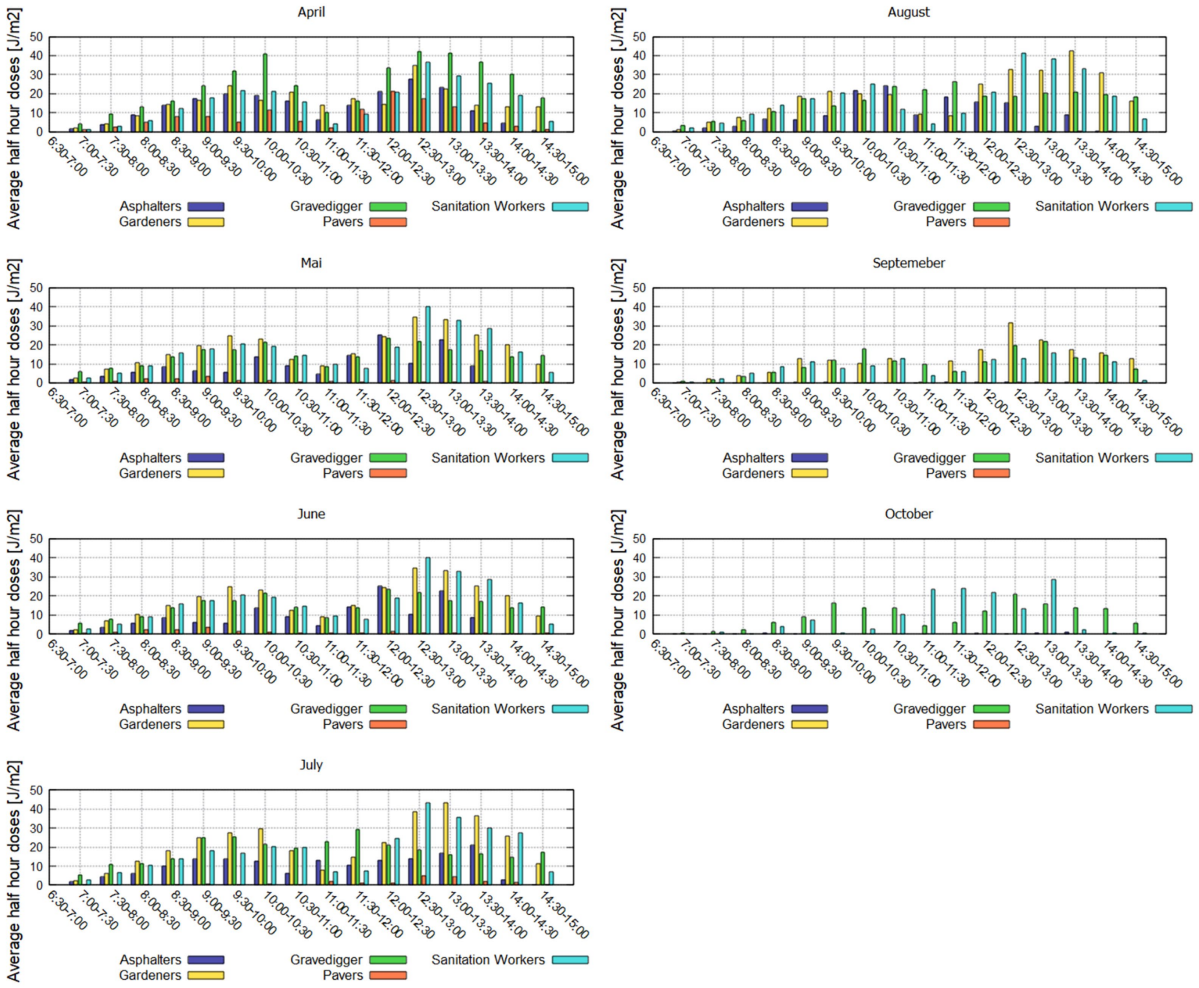
Average half-hour doses [Jm^−2^] for all measurement months for the five occupations.

Half-hour doses visualize the course of UVR exposure during the day. For that purpose measurement data are aggregated to half-hours. Figure 8 shows the average half-hour values for all occupations for each measurement month. Missing bars for some of the occupations in specific measurement months indicate that for the specific month there was no valid measurement data available.

Most of the participants seem to have a break between 11 and 12 a.m. UCT, where UVR exposure is substantially lower than expected compared with UVR levels over the course of the day. Most of the participants show higher exposure values in the afternoon (from 12 p.m.) than in the morning (before 11 p.m.). For April and May individual doses for Gravediggers are comparable in the morning and in the afternoon. Furterhmore Gravediggers show the highest half-hour doses in the months of April and May. For the rest of the measurement period (June to October), Sanitation Workers and Gardeners show the highest half-hour doses. The highest half-hour values occur in the month of July between 12:30 and 13:30 for Sanitation Workers and Gardeners (each appr. 43 Jm^−2^).

## 4 Discussion

To the best of our knowledge, our study provides the first longitudinal estimates for personal exposure to solar UVR among outdoor workers in Portugal. We conducted a measurement campaign resulting in more than 1,000 daily valid UV measurement days for six different occupations over a 7 month-period across different geographic locations in Lisbon. As expected, we showed high UVR doses cumulatively received by these workers. Long-term chronic exposure is a major risk factor for the occurrence of adverse eye and skin effects, such as cataract and skin cancers. The UVR exposure levels we identified support the urgent implementation of proper occupational health surveillance programs for the exposed workers as well as the design of tailored preventive interventions to mitigate the risk they are encountering (41, 42).

The results of this study showed that 80% of the measurement days surpass the estimated exposure limit value discussed by the ICNRP (43). The results presented are aligned with similar studies assessing the occupational UVR exposure of outdoor workers, which have reported a wide range of exposures and different patterns according to the occupations studied and locations (36). A study on construction workers in Tirgu-Mures, Romania, reported daily averages ranging from 204.5 J/m^−2^ (in October) to 680.5 Jm^−2^ (in April) (26). A multi-center study conducted across countries in Europe also identified alarming exposures for masons: average daily UVR doses ranged 148.4 Jm^−2^ to 680.5 Jm^−2^ in Romania, 342.4 Jm^−2^ to 640.8 Jm^−2^ in Italy, 165.5 Jm^−2^ to 466.2 Jm^−2^ in Croatia, 41.8 Jm^−2^ to 473.8 Jm^−2^ in Denmark and 88.2 Jm^−2^ to 400.2 Jm^−2^ in Germany (44).

In the studies that employed personal dosimeters, it is important to acknowledge that sub-occupations and specific activities, as opposed to merely job titles, serve as determining factors for UVR exposure (45). The fact that the measurements of solar UVR were conducted at the largest municipality of Portugal might also have impacted the results. Lisbon Municipality workers have access to personal protective equipment during their work hours/ duties that they can choose how to use, including long and short-sleeves t-shirts, caps and brimmed hats, and since 2023 outdoor workers have acess to sunscreen. Furthermore, certain occupations are granted a certain degree of autonomy in planning their work according to climatic conditions. The values identified in the occupation of Gravediggers are higher, primarily due to the fact that the majority most of their occupational tasks are inherently conducted in the absence of shaded shelters. The practice of preparing the grave before funeral ceremonies is a common occurrence, particularly during periods of high sun exposure. This often occurs without the presence of any shade. During funeral ceremonies, these are held in large spaces, devoid of vegetation, with minimal use of trees and consequently a paucity of shadows. Pavers were the group of individuals who registered the lowest exposure values. This phenomenon can be attributed to the fact that they may exploit the shadows present on sidewalks and squares, in order to avoid greater sun exposure. Additionally, Pavers supervisor’s have the ability to determine the work schedule for each team member, allocating them to either the morning or afternoon shift, contingent upon the availability of shaded areas. Of particular interest is the finding that Gravediggers and Pavers are the two occupations that exhibit the highest intervariability of exposure.

Sailors were excluded from the analysis due to the fact that, although three were recruited and two were enrolled, just one sailor used the dosimeter. Furthermore, the number of valid days of measurements was too low (7 days in the whole, 5 in July and one each in June and October) to be included in the comparison with other occupations.

It is important to discuss the conditions and parameters of sun exposure, encompassing the presence of shaded areas. It is aknowledged that pivotal factors influence solar UVR exposure, including geographic location, the time of the day, seasonal variations, weather conditions and the presence of reflective surfaces. The intensity of the UVR per hour of the day should also play a role, but this data is not yet available for Portugal and it is currently being prepared to more accurately describe the results of this study. The relationship between the exposure estimates obtained from the personal electronic dosimeters and the data collected by the UVR biometer is anticipated and consistent with the findings of other studies (42).

Some data seems to indicate a lunch break which was possibly spent in a shady area, e.g., at the inside of a building or a vehicle, but further investigations of the data collected within the project are needed to ensure the development of targeted health promotion interventions. A better knowledge or rather deeper understanding of the solar UVR doses of outdoor workers shall flow not only into better occupational safety measures, but also to a more advanced general protection of outdoor workers from developing occupational skin cancer by solar UVR. The study data outlines the necessity of mapping outdoor works tasks and exposure patterns and observational annotation to improve the prevention of occupational solar UVR exposure among outdoor workers in Portugal.

## 5 Conclusion

The ICNRIP occupational exposure limit value of 133 J/m^−2^ as maximum daily dose—including both occupational and recreational exposure—was surpassed in all occupations except the Pavers. Personal hourly and daily doses measured by GENESIS-UV were lower than the solar irradiation measured on a horizontal surface by the UVR biometer radiometer. The study also highlights trends in UVI in relation to the Ozone layer thickness over several decades for Lisbon. These trends indicate a decrease in total ozone related with an increase in measured UV indexes. These fluctuations will prospectively have implications that will turn solar UVR protection even more important. From the occupational and public health perspective, it is important to continue to understand how these results correlate with necessary changes in occupational safety regulations and public health awareness campaigns.

## Data Availability

The raw data supporting the conclusions of this article will be made available by the authors, without undue reservation.
