# Supplementary material for: Occupational exposure to solar ultraviolet radiation among outdoor workers in Lisbon, 2023—first results of the MEAOW study
Source: Front Public Health. 2025 Oct 27;13:1659663. doi: 10.3389/fpubh.2025.1659663 (PMC12598030; doi:10.3389/fpubh.2025.1659663)
Supplement: Supplementary file 1 [file Table_1.DOCX]

Supplementary Material

**Monthly daily averages of solar UV doses**

Table 1: Monthly daily averages of solar UV doses.

|  | **April** | | | **May** | | | **June** | | | **July** | | |
| --- | --- | --- | --- | --- | --- | --- | --- | --- | --- | --- | --- | --- |
|  | **Number of valid measurement days** | **Monthly daily average (Jm^-2^)** | **Standard**  **Deviation**  **(Jm^-2^)** | **Number of valid measurement days** | **Monthly daily average (Jm^-2^)** | **Standard**  **Deviation**  **(Jm^-2^)** | **Number of valid measurement days** | **Monthly daily average (Jm^-2^)** | **Standard**  **Deviation**  **(Jm^-2^)** | **Number of valid measurement days** | **Monthly daily average (Jm^-2^)** | **Standard**  **Deviation**  **(Jm^-2^)** |
| **Asphalters** | 72 | 192,06 | 104,14 | 51 | 188,06 | 103,17 | 15 | 195,94 | 190,18 | 8 | 294,8 | 88,17 |
| **Gardeners** | 18 | 219,83 | 138,93 | 92 | 274,5 | 126,58 | 59 | 284,48 | 107,39 | 50 | 334,88 | 188,37 |
| **Gravediggers** | 69 | 363,69 | 187,5 | 102 | 327,67 | 160,89 | 67 | 199,09 | 107,74 | 34 | 275,99 | 104,01 |
| **Pavers** | 8 | 176,17 | 163,43 | 21 | 120,64 | 101,74 | 6 | 25,62 | 20,58 | 6 | 47,58 | 109,9 |
| **Sanitation workers** | 75 | 246,71 | 162,24 | 97 | 257,24 | 153,01 | 60 | 266,91 | 179,86 | 57 | 320,62 | 238,92 |

|  | **August** | | | **September** | | | **October** | | |
| --- | --- | --- | --- | --- | --- | --- | --- | --- | --- |
|  | **Number of valid measurement days** | **Monthly daily average (Jm^-2^)** | **Standard**  **Deviation**  **(Jm^-2^)** | **Number of valid measurement days** | **Monthly daily average (Jm^-2^)** | **Standard**  **Deviation**  **(Jm^-2^)** | **Number of valid measurement days** | **Monthly daily average (Jm^-2^)** | **Standard**  **Deviation**  **(Jm^-2^)** |
| **Asphalters** | 2 | 202,62 | 36,56 | 2 | 1,33 | 0,04 | 0 | - | - |
| **Gardeners** | 40 | 298,27 | 152,55 | 6 | 187,01 | 62,4 | 0 | - | - |
| **Gravediggers** | 24 | 242,62 | 291,11 | 17 | 192,65 | 95,31 | 5 | 152,6 | 51,97 |
| **Pavers** | 7 | 1,58 | 0,61 | 1 | 2,45 | - | 0 | - | - |
| **Sanitation workers** | 52 | 276,91 | 136,83 | 10 | 152,78 | 122,61 | 1 | 134,64 | - |
